# Correlation of nipple eczema in pregnancy with atopic dermatitis in Northern India: a study of 100 cases^☆^^☆☆^

**DOI:** 10.1016/j.abd.2018.10.004

**Published:** 2019-09-30

**Authors:** Anita Puri, Anisha Sethi, Karan Jit Pal Singh Puri, Anmol Sharma

**Affiliations:** a Department of Obstetrics and Gynecology, Government Medical College and Hospital, Amritsar, Punjab, India; b Department of Skin and Venereal Diseases, Government Medical College and Hospital, Chandigarh, India; c Department of Orthopedics, Government Medical College and Hospital, Chandigarh, India

**Keywords:** Dermatitis, atopic, Eczema, Nipples

## Abstract

**Background:**

Nipple eczema is a less common presentation of atopic dermatitis. No studies in the literature have correlated nipple eczema in pregnancy as a manifestation of atopic dermatitis.

**Objective:**

To evaluate whether nipple eczema presenting in pregnancy is a manifestation of atopic dermatitis.

**Methods:**

This was a prospective observational study including 100 women who presented with nipple eczema for the first time during pregnancy. The exclusion criteria were any patient with previous history of nipple eczema, those already on oral or topical treatment for atopic dermatitis or nipple eczema, and other disorders mimicking eczema. Patients were divided into two groups ‒ nipple eczema with atopic dermatitis and without atopic dermatitis. Demographic data, clinical features, total leukocyte count, differential leukocyte count, absolute eosinophil counts, and serum IgE levels were compared between the two groups to detect association between nipple eczema in pregnancy and atopic dermatitis.

**Results:**

Out of 100 patients, 39 were diagnosed with atopic dermatitis, whereas 61 were ruled out to have any features suggestive of atopic dermatitis. There were no statistically significant differences in mean age, mean duration of symptoms, and serum IgE levels. In patients with atopic dermatitis, bilateral symptoms were noted more commonly than in patients without the disease, but this was statistically insignificant.

**Study limitations:**

Lack of long term follow-up and no large studies in literature to compare results.

**Conclusion:**

Nipple eczema in pregnancy follows a similar pattern as in other age groups. The clinical profile of patients is similar in cases with and without atopic dermatitis.

## Background

Nipple eczema is a dermatosis manifesting in many forms, such as erythema, vesicles, erosions, crusting, or fissures in the acute stage, and scaling or lichenification in the chronic stage.^1^, ^2^, ^3^ It is generally considered as a minor manifestation of atopic dermatitis (AD) but it may also be seen in other disorders, or even as a non-specific skin symptom.^4^, ^5^, ^6^ Currently, nipple eczema is known to predominate in adolescent girls and is regarded as a localized variant of AD.^7^, ^8^ AD, being the most common gestational dermatosis, has accounted for 36–49% of all pregnancy dermatoses in several studies.^9^, ^10^, ^11^, ^12^ Although nipple eczema is a less common presentation of AD, only a few studies in literature have correlated nipple eczema in pregnancy with AD or other dermatoses.

## Objectives

The objective of this study was to evaluate whether nipple eczema presenting in pregnancy is a manifestation of AD.

## Methods

The present study was a prospective observational study carried out at a tertiary care referral and teaching hospital in Northern India between 2011 and 2014, and included 100 pregnant women who presented to the OPD of the institution with the chief complaint of nipple eczema. Exclusion criteria were any patient with previous history of nipple eczema, already on oral or topical treatment for AD/nipple eczema, and other disorders mimicking eczema, such as impetigo, cellulitis, mastitis, mammary candidiasis, and Paget's disease. Approval of this study was received from the ethics committee of the institution and informed consent was obtained from each patient for participation in the study. Out of 117 consecutive patients who fulfilled the above criteria, 17 refused to volunteer and 100 were included.

Any pregnant patient presenting with the chief complaint of nipple eczema for the first time was asked about a detailed history suggestive of AD as per the diagnostic criteria proposed by Hanifin and Rajka (Table 1).^4^ Patients were thereby divided into two groups – nipple eczema with AD (Group I) and those without AD (Group II). All patients were investigated for total leukocyte counts (TLC), differential leukocyte counts, absolute eosinophil counts, and serum IgE levels. Patients in Group II were further evaluated with skin patch testing to check for any evidence of allergic contact dermatitis as a cause of eczema. Demographic data were collected and clinical features were compared between the two groups, including mean age, parity, trimester of pregnancy, duration of eczema, laterality (uni/bilateral involvement), TLC, relative and absolute eosinophil counts, and serum IgE levels. Results were analyzed and compared between the two groups to check for association between nipple eczema in pregnancy and AD.

**Table 1:** Hanifin and Rajka diagnostic criteria for atopic dermatitis (AD)

Major criteria (3 or more required)	Minor criteria (3 or more required)
Pruritus	Xerosis
Typical morphology and distribution • Flexural lichenification or linearity in adults • Facial and extensor involvement in infants and children	Ichthyosis, palmar hyperlinearity, or keratosis pilaris Immediate (type 1) skin-test reactivity Raised serum IgE
Chronic or chronically-relapsing dermatitis	Early age of onset
Personal or family history of atopy (asthma, allergic rhinitis, atopic dermatitis)	Tendency toward cutaneous infections (especially *S. aureus* and herpes simplex) or impaired cell-mediated immunity
	Tendency toward non-specific hand or foot dermatitis
	Nipple eczema
	Cheilitis
	Recurrent conjunctivitis
	Dennie-Morgan infraorbital fold
	Keratoconus
	Anterior subcapsular cataracts
	Orbital darkening
	Facial pallor or facial erythema
	Pityriasis alba
	Anterior neck folds
	Itch when sweating
	Intolerance to wool and lipid solvents
	Perifollicular accentuation
	Food intolerance
	Course influenced by environmental or emotional factors
	White dermographism or delayed blanch

A hypothesis formulated before data collection was that nipple eczema presenting for the first time in pregnancy is a manifestation of AD. Statistical analysis was performed using Fisher's exact test, the chi-squared test, or the *t*-test as appropriate, and *p*-values less than 0.05 were regarded to be statistically significant.

## Results

The present study included 100 cases diagnosed as nipple eczema for the first time in pregnancy. The patients were divided into two groups – nipple eczema with AD (Group I, *n* = 39) and without AD (Group II, *n* = 61). The demographic and clinical characteristics of both the groups are shown in Table 2. There was no statistically significant difference in mean age, mean duration of symptoms, and serum IgE levels between the two groups. In both groups, presentation for the first time was most commonly noted in second trimester of pregnancy. In patients with AD, bilateral symptoms were noted more commonly (79.4% cases) than in patients without AD (63.9%), but this difference was not found to be statistically significant. Differential eosinophil counts and absolute eosinophil counts were observed to be significantly higher in the group with AD. Out of the total 61 cases of nipple eczema who were ruled out for AD (Group II), patch testing showed 11 (18.03%) cases to be positive for allergic contact dermatitis using skin patch tests, which included potassium bichromate (four cases), most commonly followed by wool alcohol, chromium sulfate, and nickel, in that order. Soap was found to be the most common aggravating factor.

**Table 2:** Clinical characteristics

	Nipple eczema with AD (Group I) (*n* = 39)	Nipple eczema without AD (Group II) (*n* = 61)	*p*-Value
Mean age (years)	27.4	28.6	0.07
Parity	P1 = 35.9%	P1 = 39.4%	0.87
	P2 = 53.8%	P2 = 50.8%	
	>P2 = 10.3%	>P2 = 9.8%	
Trimester of pregnancy	T1 – 28.5%	T1 – 32.1%	0.21
	T2 – 49.4%	T2 – 37.7%	
	T3 – 22.1%	T3 – 30.2%	
Duration of eczema (in weeks)	24.5	22.7	0.19
Laterality (uni/bilateral)	Bi = 79.4%	Bi = 63.9%	0.09
Total leukocyte count (per μL)	8387.1	6942.8	<0.01
Differential leukocyte count (% of eosinophils)	6.6 ± 5.1	2.1 ± 1.7	<0.01
Absolute eosinophil count/μL	392.5 ± 202.8	107.3 ± 81.6	<0.01
Serum IgE levels (IU/mL)	310.5	292.7	0.17

A possible limitation of our study was lack of long-term follow-up of patients. Patients who did not fulfill the diagnostic criteria of AD might manifest other symptoms of AD in the future. In addition, there were no large studies in literature with which to compare the results.

## Discussion

Nipple eczema, although considered to be a minor diagnostic criteria for diagnosis of AD, is one of the most common clinical presentations of AD in the breast.^1^, ^4^ Nevertheless, there have been studies that show that nipple eczema was not a characteristic finding of AD.^5^, ^6^ Although typically, nipple eczema is noted predominantly in adolescent females,^7^ its incidence in pregnancy, where AD is the most common dermatosis, deserves mention. In the present study's literature search, no studies correlating nipple eczema in pregnancy with AD could be found. In their report, Amato et al.^2^ diagnosed a patient with AD exclusively localized to the nipples and areolas with celiac disease and sensitization to nickel and thimerosal. They opined that nipple eczema should be re-evaluated as an important diagnostic sign of AD, especially during pre-puberty and breastfeeding and when it is not associated with other typical lesions of AD (morphologically and topographically). Similarly to the incidence of AD in pregnancy, nipple eczema was also most commonly manifested in first and second trimester, but in both groups. Duration of eczema before first presentation was slightly greater in patients with AD, but this finding was statistically insignificant. Nipple eczema is usually known to have a bilateral involvement. In the present study, 30% cases had unilateral involvement only, especially in cases without AD. This was similar to the findings of Song et al.^13^ Differential ratio of eosinophils and absolute eosinophil counts in blood were significantly higher in the group with AD. This was similar to the findings of other studies on AD and was probably not due to the difference in the character of nipple dermatitis between the two groups, but rather due to AD itself.^14^ Serum IgE levels were similar in both the groups; this is in agreement with the study by Song et al., who suggested that the level of total IgE or other specific IgE does not correlate with the presence of AD in patients with nipple eczema.^13^ Through clinicopathological and immunohistochemical analyses of nipple eczema with AD, they also suggested that nipple eczema might not be an indicative manifestation of AD.

A possible limitation of our study was lack of long-term follow-up of patients. Patients who did not fulfill the diagnostic criteria of AD might manifest other symptoms of AD in the future. In addition, there were no large studies in literature with which to compare the results.

## Conclusion

Nipple eczema in pregnancy follows a similar pattern as in other age groups. The clinical profile of patients is similar in cases with and without AD. Although there is a statistically significant difference in the TLC, as well as the relative and absolute eosinophil counts between the groups, this can be attributed to the presence of AD itself and not due to a difference between the two groups in the character of the nipple eczema. Thus, it can be concluded that there is no correlation between nipple eczema in pregnancy and AD.

## Financial support

None declared.

## Author's contribution

**Anita Puri:** Approval of the final version of the manuscript; conception and planning of the study; obtaining, analyzing and interpreting the data; critical review of the literature, critical review of the manuscript.

**Anisha Sethi:** Statistical analysis; approval of the final version of the manuscript; conception and planning of the study; elaboration and writing of the manuscript; obtaining, analyzing and interpreting the data; effective participation in research orientation; intellectual participation in propaedeutic and/or therapeutic conduct of the cases studied; critical review of the literature; critical review of the manuscript.

**Karan Jit Pal Singh Puri:** Approval of the final version of the manuscript; conception and planning of the study; effective participation in research orientation; intellectual participation in propaedeutic and/or therapeutic conduct of the cases studied; critical review of the literature; critical review of the manuscript.

**Anmol Sharma:** Statistical analysis; approval of the final version of the manuscript; elaboration and writing of the manuscript; obtaining, analyzing and interpreting the data; effective participation in research orientation; critical review of the literature; critical review of the manuscript.

## Conflicts of interest

The authors declare no conflicts of interest.
